# Case Report: Rare Peripheral Neuropathy With Abdominal Wall Allodynia in Extensive‐Stage Small‐Cell Lung Cancer Treated Using Durvalumab

**DOI:** 10.1111/1759-7714.70368

**Published:** 2026-07-26

**Authors:** Ruidan Li, Zhen Li, Xiaoqian Zhai, Jie Shao, Yaqin Wang, Qinghua Zhou, Jiewei Liu

**Affiliations:** ^1^ Respiratory and Critical Care Medicine Department Norinco General Hospital Xi'an Shaanxi China; ^2^ West China School of Medicine, Sichuan University Chengdu Sichuan China; ^3^ Lung Cancer Center/Lung Cancer Institute, West China Hospital Sichuan University Chengdu Sichuan China; ^4^ Department of Thoracic Surgery West China Hospital, Sichuan University Chengdu Sichuan China

**Keywords:** allodynia, durvalumab, immune‐related adverse event, lung cancer, mmune checkpoint inhibitor

## Abstract

Immune checkpoint inhibitors (ICIs), such as PD‐1/PD‐L1 inhibitors, have revolutionized the treatment of extensive‐stage small‐cell lung cancer (ES‐SCLC). Chemotherapy combined with PD‐1 or PD‐L1 inhibitors has become the standard first‐line treatment for ES‐SCLC. However, with the broad application of immunotherapy, adverse events associated with this therapy have been increasingly reported by clinicians. The identification of atypical and specific manifestations of immune‐related adverse events (irAEs) requires accumulated experience on the part of clinicians. In this article, we report the case of a patient over 50 years of age who was initially diagnosed with ES‐SCLC. Clinical symptoms mainly manifested as allodynia of the abdominal wall following PD‐L1 inhibition immunotherapy. This symptom progressed with continued immunotherapy and resolved after cessation of immunization and treatment with prednisone. A peripheral neuropathy antibody test revealed anti‐GM4 IgG antibody positivity. On the basis of the patient's clinical manifestations and medication history described above, a diagnosis of immune‐related peripheral neuropathy was made. In this case, the irAE severely hindered the treatment process and impaired the patient's quality of life. Through this case report and a review of the literature, we aim to explore how to timely and accurately identify irAEs in immunotherapy, especially nonspecific irAEs, and provide timely treatment to improve patient quality of life and survival and immunotherapy precision and safety.

## Graphical abstract

This case describes a rare allodynia irAE induced by durvalumab in a patient with ES‐SCLC. It is a case of peripheral neuropathy, but the main manifestation differs from most previous cases. First, based on CT, ECG, and cardiac biomarker results, we ruled out cancer‐related pain and organic lesions. Subsequently, a 30‐item antibody test for peripheral neuropathy revealed anti‐GM4 IgG antibody positivity. Moreover, the presence of an analgesic response to corticosteroid therapy and recurrence upon rechallenge led us to conclude that this is a peripheral neuropathy‐associated irAE. In this case, the irAE was characterized by persistent allodynia across the midline of the abdominal wall, with no skin abnormalities and rapid pain improvement after hormone therapy. However, previously reported ICI‐induced irAEs have mostly manifested as weakness, numbness, and pain at the ends of limbs.

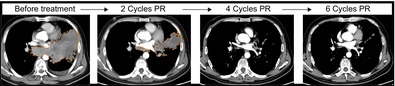

## 1 Introduction

After confirmation of the efficacy of PD‐1/PD‐L1 inhibitors by extensive clinical studies, lung cancer treatment has entered the era of immunotherapy. However, the mechanism through which immunotherapy activates the body's immune system to achieve antitumor efficacy may result in immune‐related adverse events (irAEs). Among irAEs, neurological irAEs remain rare, occurring in only 1% to 5% of patients [1]. Most immune‐related peripheral neuropathies are characterized by pain distributed in the area of the affected nerves, with weakness, numbness, and pain at the ends of the limbs [2]. However, in our case, a patient with extensive‐stage small‐cell lung cancer (ES‐SCLC) developed significant abdominal wall allodynia following treatment with durvalumab. Allodynia was suspected to be immune‐related and neuropathic on the basis of clinical features and test results; this case provides new information for the management of neurologic irAEs.

### 1.1 Case Presentation

The patient, who was over 50 years of age, presented to a doctor in June 2022 with “recurrent cough for 10+ months and hemoptysis for 8+ months”. The patient was subsequently diagnosed with SCLC of the left hilar with lymph node metastases in the left hilar and mediastinal regions, left adrenal gland metastases and multiple bone metastases (extensive‐stage, T4N3M1c, IVB1).

**FIGURE 1:**
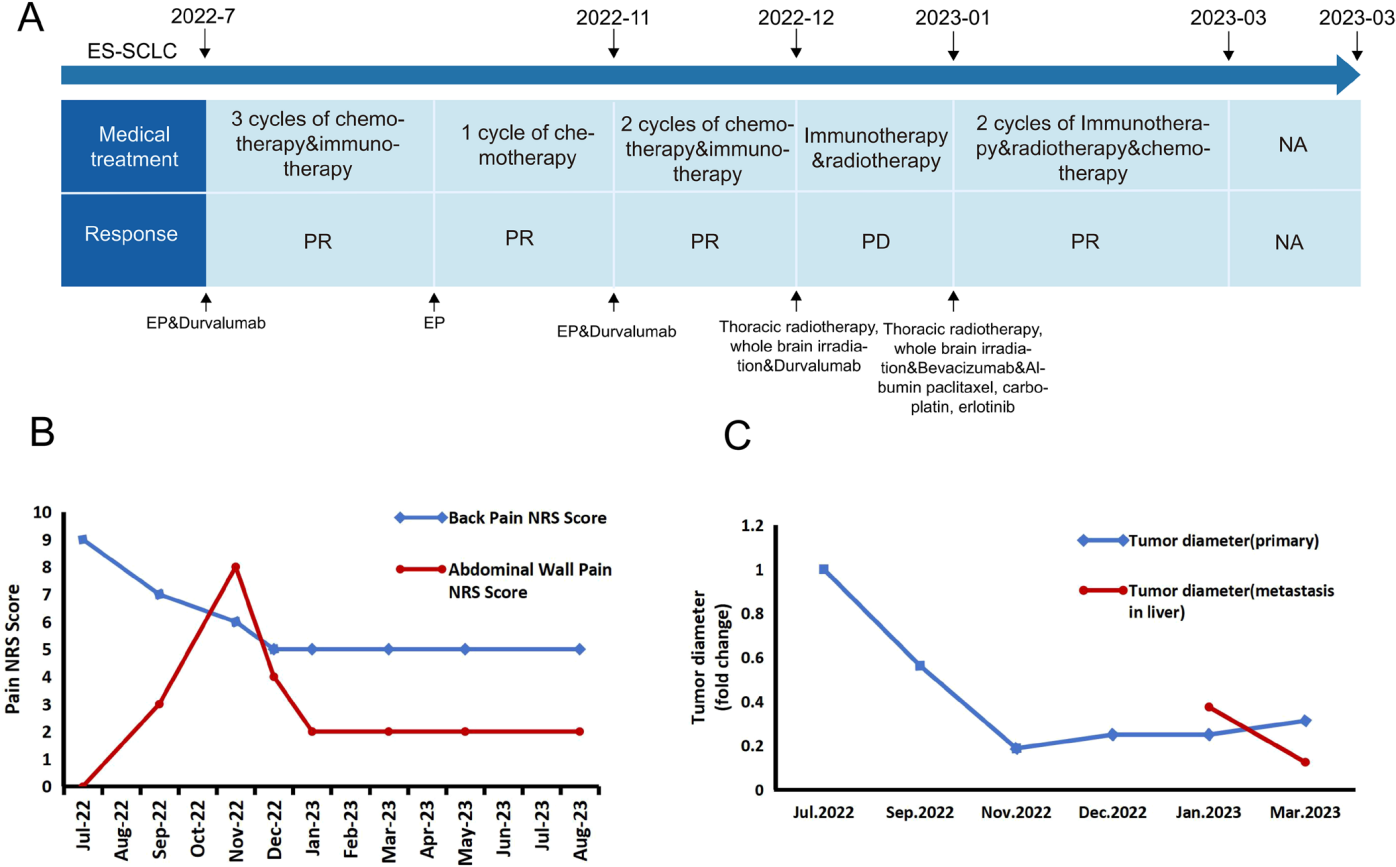
(A) The therapeutic process and overall survival response. (B) Changes in the patient's NRS score. (C) The longest diameters of the primary tumor and metastatic tumor.

At initial diagnosis, the patient experienced back pain due to vertebral metastases and bone destruction. However, oral OxyContin (40 mg every 12 h) provided adequate pain control. A partial response (PR) and significant relief of back pain were achieved after 2 cycles of etoposide and platinum (EP) combined with durvalumab. However, the patient gradually developed allodynia in the left anterior abdominal wall. This allodynia was superficial in location, characterized by persistent, burning pain that worsened with gentle touch. The affected skin area did not exhibit blisters, redness, or other manifestations of rash and was located across the midline of the abdominal wall, with no apparent role in respiratory motility, feeding, or other functions. Allodynia in this patient was not accompanied by palpitations, chest tightness, precordial pressure, or other discomforts, such as acid reflux, belching, or epigastric pressure. The temperature sensation of the impacted skin was normal. Allodynia was poorly controlled after oral administration of 40 mg of OxyContin q12h for pain relief. Furthermore, allodynia worsened significantly after the third cycle of treatment with durvalumab, and the NRS score reached 8 (Figure 1B).

To explore the etiology of allodynia in this patient, chest computed tomography (CT), abdominal CT, electrocardiography (ECG) and cardiac biomarker assays were performed. As all results were unremarkable, neoplastic etiologies and organic lesions were excluded. A 30‐item antibody test for peripheral neuropathy was completed, revealing anti‐GM4 IgG antibody positivity. In addition, the levels of the inflammation‐related cytokines IL‐1β, IL‐2, IL‐4, IL‐6, IL‐8, TNFα, G‐CSF, and ferritin were greatly increased. The patient's allodynia was rapidly relieved with oral prednisone (40 mg qd) for 3 days, followed by a tapering regimen consisting of a 5 mg reduction every 3 days, with a total treatment duration of 21 days. Given the irAEs caused by durvalumab, the 4th infusion of durvalumab was suspended, and allodynia did not recur. Moreover, negative autoantibody test results excluded the presence of primary autoimmune disorders. Therefore, we hypothesized that the allodynia in this patient may have been induced by durvalumab‐associated immune disorder. Although the patient refused further examination by electromyography, the parallel incidence and disappearance of allodynia and durvalumab application validated this hypothesis.

**FIGURE 2:**
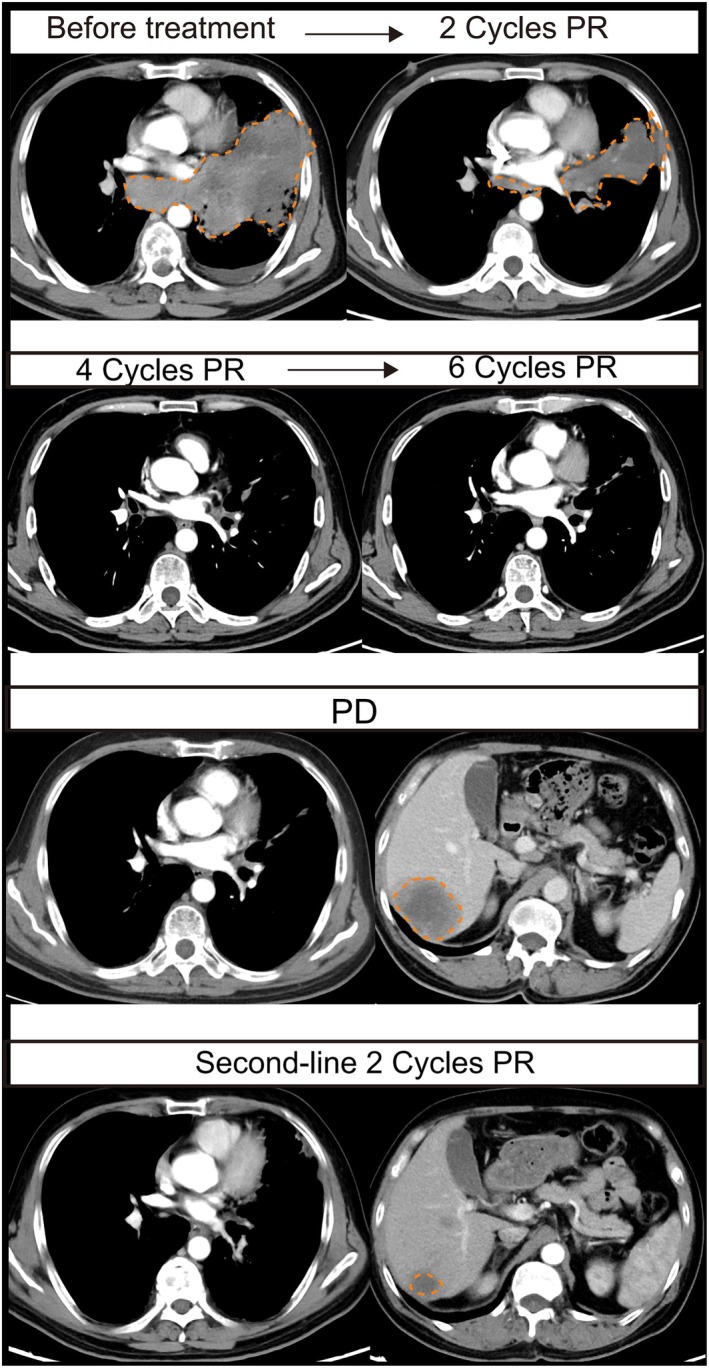
Enhanced CT images of primary tumors and metastatic tumors in the liver highlighted with yellow circles.

To further control disease progression, an MDT discussion allowed the reuse of immunotherapy in cycle 5 on the basis of the confirmed efficacy (partial response (PR) after 2 cycles), the absence of severe organ damage, and prior experience with safe rechallenge reported by Chye et al. [3]. Two days after the completion of immunotherapy, the patient experienced recurrent abdominal wall pain characterized by persistent tenderness crossing the midline of the abdominal wall, although the intensity was significantly alleviated compared with that during previous episodes. The pain became tolerable after oral administration of 40 mg of OxyContin every 12 h and did not recur despite corticosteroid injection therapy; the NRS score was 3. After 6 cycles of chemotherapy combined with immunotherapy, the patient continued to receive durvalumab immuno‐maintenance therapy, thoracic radiotherapy (50 Gy/25f), and prophylactic whole‐brain irradiation of the head (25 Gy/10f), resulting in stable disease control. Progressive disease (PD), presenting as an enlarged liver and adrenal and lymph node metastases, occurred after 6 months of first‐line treatment (Figures 1C and 2). Thus, second‐line treatment with albumin paclitaxel plus carboplatin in combination with anlotinib was initiated. Moreover, durvalumab treatment continued, and PR was achieved again after 2 cycles. During second‐line antitumor therapy, the patient, who was receiving 40 mg of OxyContin q12 orally, maintained a NRS score of ≤ 3. However, because of chemotherapy intolerance (nausea, vomiting, and poor appetite), the patient was switched to anlotinib in combination with immune maintenance therapy, and pain control was stable. During third‐line antitumor therapy and until the end of life, the dosage of OxyContin was adjusted to 60 mg q12 h to improve patient's pain control and maintain their quality of life, with the NRS score remaining ≤ 3 (Figure 1B). After 6 months, the patient entered the end stage because of tumor progression, with an overall survival of 16 months (Figure 1A).

## 2 Discussion

This case highlights a rare irAE following durvalumab treatment in a patient with ES‐SCLC. This rare irAE presented as severe pain localized to the epidermis of the abdominal wall, which was characterized by persistent allodynia across the midline of the abdominal wall, with no abnormality in the skin and rapid improvement in pain after hormone therapy. Pain in this patient was significantly associated with durvalumab administration, as the symptoms resolved following immunotherapy discontinuation and corticosteroid therapy, and primary autoimmune disorders were excluded.

Notably, when the patient reported allodynia of the abdominal wall, anti‐GM4 IgG antibodies were detected in the blood. While peripheral neuropathy is typically linked to anti‐GM1, anti‐GM2, or anti‐GM3 IgG antibodies [4], anti‐GM4 IgG antibodies selectively bind the spinal dorsal root and autonomic ganglia [5, 6], triggering localized immune activation and hyperalgesia. Given the superficial location and weak barrier of the abdominal wall ganglia, this mechanism explains the observed localized pain, which contrasts with generalized polyneuropathy mediated by antibodies against GM1, GQ1b, and others. Taken together, the above evidence demonstrates that this neurologic irAE was caused by durvalumab. In our case, allodynia occurred after 2–3 cycles of durvalumab, the timing of which was consistent with the median duration of usual neurologic irAE occurrence [7]. To further distinguish our case from other kinds of pain, the main differences between tumor‐related pain, treatment‐related pain, and other types of neuropathic pain, such as herpes zoster, which may occur in tumor patients, are listed in Table 1.

**TABLE 1:** Classification and differential diagnostic points of tumor‐associated pain.

Type of pain	Pain localization	Nature of pain	Relationship with anti‐tumor drugs	Mitigating drug
Tumor invasion (cancer pain)	Location of the tumor	Injury pathology	Antitumor therapy provides relief	Anti‐tumor drugs
Due to chemotherapeutic agents (e.g., paclitaxel analogs)	Extremities are predominantly extremities	Neuropathic	Antitumor therapy may exacerbate	Non‐opioid
Immune‐related	Changeable	Neuropathic	Antitumor therapy may exacerbate	Hormones
Shingles	Epidermis, not exceeding the midline of the body	Neuropathic	Possible reduction, but non‐specific, other generalized discomfort may occur	Antiviral therapy, neurotrophic

Compared with previous cases of irAEs following PD‐L1 inhibitor therapy, our case is unique. We studied 13 previous cases of lung cancer with peripheral neuropathy as the primary irAE after the use of ICI. Peripheral neuropathy in these cases manifested mostly as weakness, numbness, and pain at the ends of the limbs (Table 2). Conversely, our patient's pain was mainly characterized by persistent allodynia across the midline of the abdominal wall, with no abnormalities in the skin and rapid improvement in pain after hormone therapy. Furthermore, the finding that hormonal therapy was effective in this type of irAE provides a new basis for identifying and managing atypical clinical manifestations of neurological irAEs.

**TABLE 2:** Summary of the characteristics of immune‐associated peripheral neuropathy and its treatment in lung cancer patients undergoing immunotherapy.

References	Age	Histological type	Staggered	Treatment program	Immunization drug	Clinical manifestation	Therapeutic measure
Ding et al. [8]	50+	Squamous carcinoma	IV	Chemotherapy + immunotherapy	KN046	Muscle weakness and numbness in the limbs	Glucocorticoids + gamma globulin + mycophenolate mofetil
Ao et al. [9]	50+	Adenocarcinoma	cT4N3M0, IIIC	Chemotherapy + immunotherapy	Sindilizumab	Numbness and weakness of the limbs, lower limbs obviously Difficulty in standing after squatting	Glucocorticoids + pyridoxamine bromide tablets
Ao et al. [9]	60+	Small‐cell neuroendocrine carcinoma	IV	Chemotherapy + immunotherapy	Teraplizumab	Weakness and numbness in the limbs	Glucocorticoids
Li et al. [10]	60+	Adenocarcinoma	cT3N0M1a, IVA	Chemotherapy (docetaxel) + immunotherapy	Tirelizumab	Weakness, numbness, and pain in the limbs	γ‐globulin + acupuncture
Yamanaka et al. [11]	70+	Small‐cell lung cancer	cT1cN3M1a, IVA	Chemotherapy (etoposide + carboplatin) + immunotherapy	Atilizumab	Weakness in the distal joints of the fingers and feet of both hands	Glucocorticoids + γ‐globulin
Oguri et al. [12]	70+	Adenocarcinoma	cT2bN2M1c, IVB	Radiotherapy + immunotherapy	Pembrolizumab	Pain and weakness in joints and muscles of the limbs	Glucocorticoids + γ‐globulin
Kurashige et al. [13]	50+	Atypical carcinoid tumor	cT1bN2M1c, IVB	Immunotherapy	Pembrolizumab	Blood in stool, muscle weakness, and pain in limbs	Glucocorticoids + infliximab + rectal resection
Mazzaschi et al. [14]	80+	Adenocarcinoma	IV	Chemotherapy (gemcitabine, pemetrexed) + immunotherapy	Navulizumab	Progressively increasing weakness and pain in the lower extremities, numbness in the distal upper extremities, and inability to perform fine motor skills	Glucocorticoids + immunoglobulins
Nukui et al. [15]	40+	Metastatic lung cancer (nasal cancer)	IV	Immunotherapy	Navulizumab	Weakness of both eyelids and numbness of the extremities	Immunoglobulins
Fukumoto et al. [16]	60+	Non‐small‐cell lung cancer	IV	Chemotherapy (carboplatin + paclitaxel) + immunotherapy	Navulizumab	Severe weakness and abnormal sensation in the distal extremities	Immunoglobulins
Thapa et al. [17]	60+	Adenocarcinoma	IV	Chemotherapy (carboplatin + pemetrexed) + immunotherapy	Navulizumab	Distal upper extremity and proximal lower extremity weakness evident	Glucocorticoids + immunoglobulins
Pomerantz et al. [18]	50+	Small‐cell lung cancer	IVB	Anrotinib + immunotherapy	Navulizumab‐ipilimumab	Abnormal sensation, pain, weakness, or even inability to walk in the lower limbs	Immunoglobulins

To our knowledge, this is the first case of peripheral neuropathy with allodynia in the abdominal wall as an irAE manifestation during durvalumab treatment. This irAE severely impaired the patient's quality of life and treatment process. The clinical presentation was atypical, and corticosteroid therapy was effective at managing this type of irAE. These findings provide new insights into the differentiation and management of clinical manifestations of immune‐related neurotoxicity. Our case also serves as a novel source of information related to irAEs to assist oncologists in such situations.

In terms of limitations, in this case, owing to the severity of abdominal wall pain (an NRS score of 8) and the consequent urgent psychological desire for pain relief, the patient refused key electromyography (EMG) examination, and these important objective data are unavailable, which represents a major limitation of this study. However, anti‐GM4 IgG antibody positivity, negative CT for irAEs, and steroid responsiveness confirm this case as a distinct type of neuropathic ICI toxicity.

## Author Contributions

**Zhen Li:** writing – review and editing. **Jie Shao:** writing – original draft. **Jiewei Liu:** writing – review and editing. **Ruidan Li:** writing – original draft, project administration. **Qinghua Zhou:** writing – review and editing. **Xiaoqian Zhai:** project administration, writing – review and editing, funding acquisition. **Yaqin Wang:** writing – original draft.

## Funding

This work was supported by National Natural Science Foundation of China (82503629 [X.Z.]). The Sichuan Provincial Natural Science Fund (2024NSFSC1929 and 2024NSFSC0743 [X.Z.]). The Science and Technology Bureau of Chengdu (2026‐YF11‐00034‐HZ [X.Z.]). Guangdong Association of Clinical Trials (GACT)/Chinese Thoracic Oncology Group (CTONG) and Guangdong Provincial Key Lab of Translational Medicine in Lung Cancer (Grant no. 2017B030314120).

## Ethics Statement

This study was approved by the Ethical Research Committee of the West China Hospital (2021–264).

## Consent

Written informed consent was obtained from the patient to publish the case report along with all accompanying visual elements.

## Conflicts of Interest

The authors declare no conflicts of interest.

## Data Availability

The data that support the findings of this study are available from the corresponding author upon reasonable request.
